# Multi-omics analysis identifies potential mechanisms of AURKB in mediating poor outcome of lung adenocarcinoma

**DOI:** 10.18632/aging.202517

**Published:** 2021-02-17

**Authors:** Jie Huang, Qianyun Zhang, Juan Shen, Xueqin Chen, Shenglin Ma

**Affiliations:** 1 Nanjing Medical University, Nanjing, China; 2 Department of Oncology, Nanjing Medical University Affiliated Hangzhou Hospital, Hangzhou, China; 3 Department of Oncology, Key Laboratory of Clinical Cancer Pharmacology and Toxicology Research of Zhejiang Province, Affiliated Hangzhou First People’s Hospital, Zhejiang University School of Medicine, Hangzhou, China

**Keywords:** AURKB, lung adenocarcinoma, prognosis, methylation, microRNAs

## Abstract

Aurora kinases B (AURKB), which plays a critical role in chromosomal segmentation and mitosis, greatly promotes cell cycle progression and aggressive proliferation of cancers. So far, its role and underlying mechanisms in mediating poor outcome of lung adenocarcinoma (LUAD) remained largely unclear. Analyses on multiple omics data of lung adenocarcinoma cohort in The Cancer Genome Atlas (TCGA) were performed based on AURKB expression, and demonstrated its association with clinical characteristics and the potential of using AURKB as a biomarker in predicting patients’ survival. This study found aberrant alterations of genomics and epigenetics, including up-regulation and down-regulation of oncogenic genes and tumor suppressors, pathways involved in the cell cycle, DNA repair, spliceosome, and proteasome, hypermethylation enrichments around transcriptional start sites, which are all related to AURKB expression. We further discovered the possible role of tumor suppressors DLC1 and HLF in AURKB-mediated adverse outcome of LUAD. To conclude, this study proved AURKB as a potential prognostic factor and therapeutic target for lung adenocarcinoma treatment and provide a future research direction.

## INTRODUCTION

Lung adenocarcinoma (LUAD) is the main type of lung cancer. Although epidermal growth factor receptor (EGFR) tyrosine kinase inhibitors (TKIs) [1] and programmed death-ligand 1/programmed death 1 (PD-L1/PD1) targeting immunotherapy used in combination with or without chemotherapy has been successful [2], the underlying mechanisms leading to poor survival or even unresponsiveness to these therapies in patients with lung adenocarcinoma remain to be elucidated. Aurora kinases B (AURKB), a key modulator of chromosome segregation in mitosis, plays an important role in proliferation and metastasis in many cancers. For instance, the expressions of AURKB and Aurora kinases A (AURKA) have been found to be upregulated by Myc and essential in Myc-driven B-cell lymphomas [3]. Barasertib is effective in the discontinuation of AURKB for the treatment of pediatric acute leukemia [4], and AURKB is also detected in many solid tumors, such as gastric cancer [5, 6], glioblastoma [7], bladder cancer [8], and clear cell renal cell carcinoma [9]. As early as 2005, AURKB is found to be correlated with high frequencies of somatic mutations in lung cancer [10]. Researches showed that AURKB is an important target for KRAS, therefore has the potential to serve as a target of KRAS-induced lung cancer [11]. Recently, study discovered that AURKB is responsible for cancer resistance to chemical reagents such as cisplatin and paclitaxel [12], and it has been established as a potential target to overcome the acquired resistance to EGFR TKIs when patients do not harbor resistance mutations in the EGFR gene [13]. Additionally, radiotherapy sensitivity of lung cancer could be enhanced by inhibition of AURKB induced by an herbal drug Daurinol [14]. Therefore, AURKB plays a critical part in lung cancer and may be a vital drug target to reverse the resistance to chemotherapy, targeted therapy, or radiotherapy.

Although some mechanisms of AURKB have been shown above, the important role of AURKB in lung adenocarcinogenesis and aggressiveness leading to poorer survival requires further exploration. Through analysis of the genome-wide mRNAs, microRNAs (miRNAs) and methylation profiles from LUAD project in The Cancer Genomics Atlas (TCGA) and the web-based bio-tool Lung Cancer Explorer (LCE) [15], we showed evidence for the use of AURKB as a prognosis biomarker in LUAD patients, and demonstrated potential genomic and epigenomic mechanisms associated with AURKB expression, explaining how AURKB accelerated the lung adenocarcinoma progression and limited patients’ survival.

## RESULTS

### Overexpression of AURKB in LUAD patients

**Figure 1:**
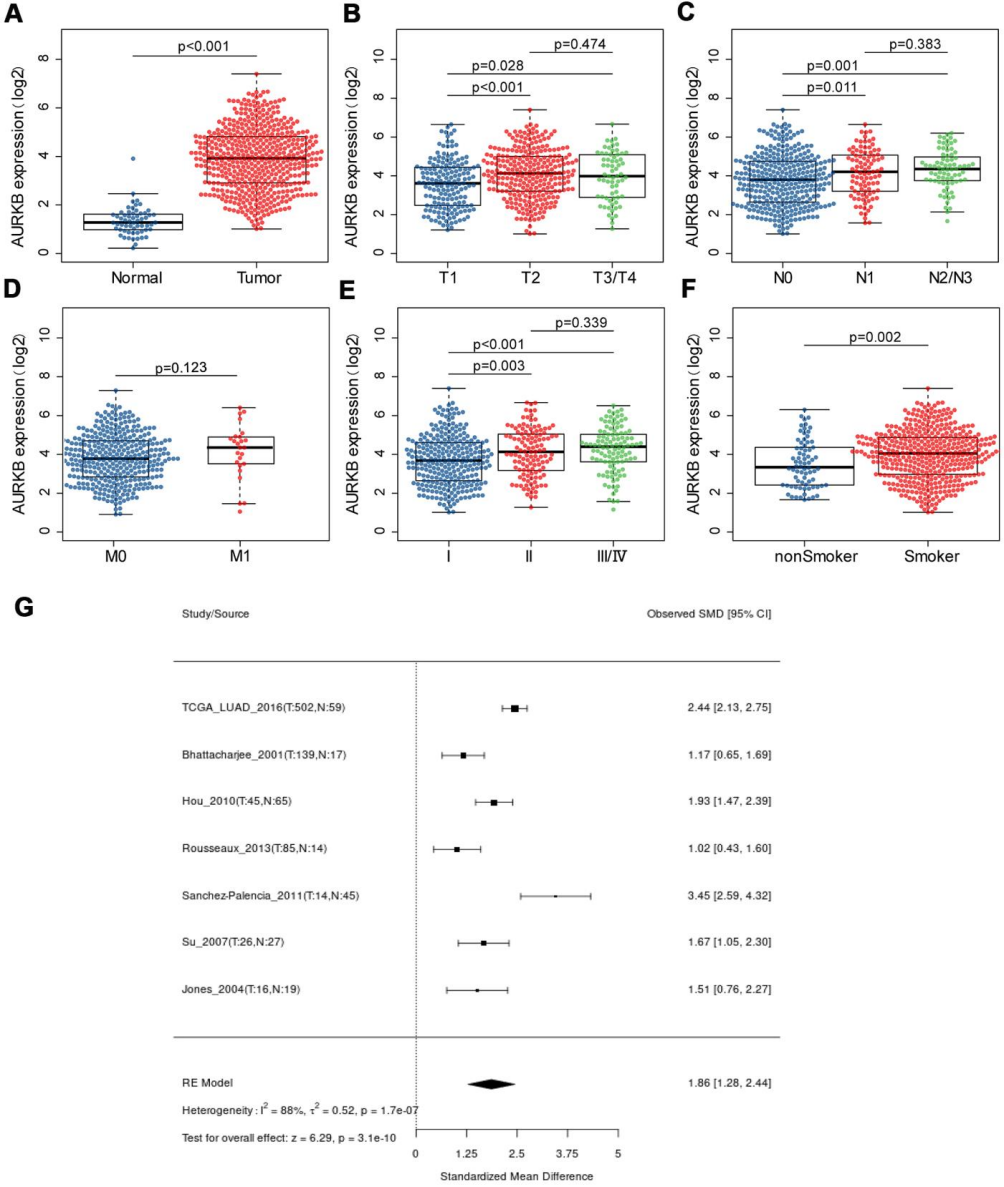
**The expression of AURKB in LUAD patients and its association with clinical characteristics.** (**A**) AURKB expression difference between lung adenocarcinoma samples and normal samples; (**B**–**E**) AURKB expression in different subgroups characterized by TNM staging system (**B**) T for tumor size; (**C**) N for lymph node invasion; (**D**) M for distant metastasis; (**E**) stage); (**F**) AURKB expression in smokers and non-smokers. (**G**) Meta-analysis of AURKB expression in tumor and normal tissues based on different datasets by Lung Cancer Explorer (LCE).

TCGA-LUAD cohort was used to compare the expression of AURKB in lung adenocarcinoma patients. As shown in Figure 1A, AURKB was overexpressed in LUAD tumor tissues (513 cases) when compared with 59 normal tissues (rank-sum test, p < 0.001). We further examined the correlation between AURKB expression and the clinical features of the 513 lung adenocarcinoma patients. For TNM system, we found that AURKB was significantly low-expressed in tumors with smaller size (T1 stage) when compared with T2 (p<0.001) or T3-4 (p=0.028) (Figure 1B). Patients without lymph node invasion (N0) showed low expression of AURKB than those with N1 (p=0.011) or N2-3 (p=0.001) (Figure 1C). No significant differences among patients with or without metastasis (p=0.123, Figure 1D) were detected, which might be due to the limited number of metastatic patients in the LUAD cohort. However, AURKB showed obvious variations in the patients’ staging: lower expression in stage I against stage II (p=0.003) or III/IV (p<0.001), as shown in Figure 1E. We also found that AURKB was noticeably high-expressed in patients with a history of smoking (p=0.002, Figure 1F), and that elder or female patients tend to have a relatively lower expression of AURKB (p=0.041, 0.0028, respectively, as shown in Table 1). We additionally compared 7 microarrays and sequencing data sets using the lung cancer explorer (LCE) database, and the results confirmed that AURKB expression was up-regulated in lung adenocarcinoma (p=3.1e-10, Figure 1G).

**Table 1:** Relationships between AURKB expression and clinicopathological features.

**Characteristic**	**All patients**	**AURKB ^high^**	**AURKB ^low^**	***P-*value**
Age(years)				
Mean ± SD	65.21±10.01	64.28±10.49	66.15±9.42	***0.041***
Gender				
Male	228	131	97	***0.0028***
Female	262	114	148	
Depth of invasion				
T1	163	67	96	***0.015***
T2	263	146	117	
T3+T4	61	31	30
Lymph node metastasis				
N0	317	146	171	***0.0056***
N1+N2+N3	162	97	65	
Distant metastasis				
M0	324	158	166	0.277
M1	24	15	9	
TNM stage				
I	263	114	149	***0.0022***
II	115	63	52	
III+IV	104	65	39
Smoking history				
Smoker	408	217	191	***0.0093***
Non-smoker	68	24	44	
EGFR				
Mutation	79	39	40	0.99
Non-mutation	186	90	96	

### AURKB indicates a worse outcome in LUAD

**Figure 2:**
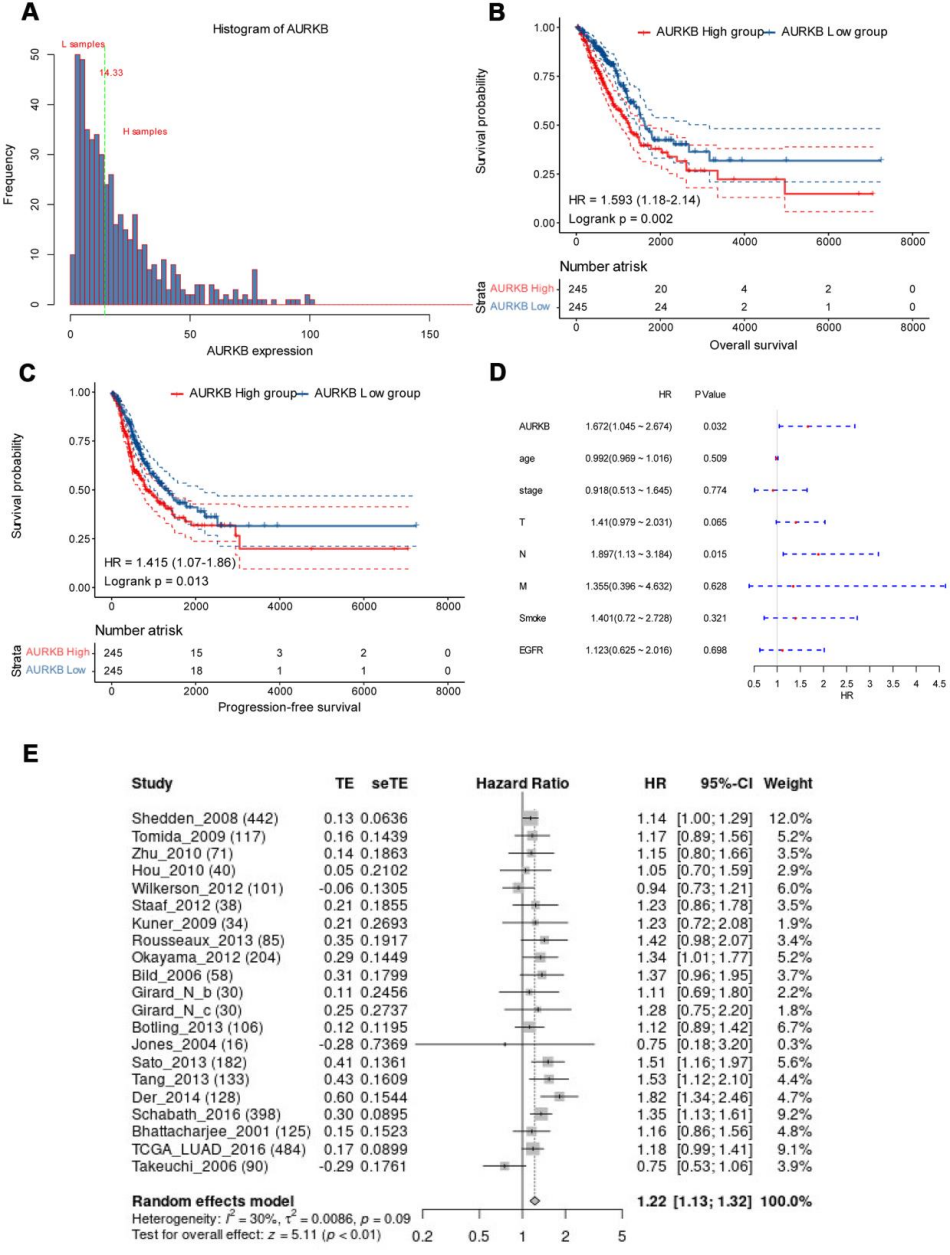
**AURKB could independently predict the survival of lung adenocarcinoma.** (**A**) AURKB expression distribution in all tumor samples; (**B**) High AURKB expression led to worse overall survival in the TCGA-LUAD cohort (HR[95%CI]: 1.593[1.18-2.14], p = 0.002). (**C**) High AURKB expression correlated with poor progression-free survival in the TCGA-LUAD cohort (HR[95%CI]: 1.415[1.07-1.86], p = 0.013). (**D**) Multivariate analysis showed AURKB and N stage were independent prognosis factor in the TCGA-LUAD cohort (HR[95%CI]: 1.672[1.045-2.674], 1.897[1.13-3.184], respectively). (**E**) Meta-analysis of AURKB expression in the prediction of overall survival of lung adenocarcinoma patients from different datasets by Lung Cancer Explorer (LCE).

As AURKB is high-expressed in lung adenocarcinoma tissues and correlates with more advanced tumor stage, we hypothesized that AURKB might be a prognostic factor in LUAD. Thus, the expression distribution of AURKB and prognosis of each sample were examined. We observed that the expression of AURKB of each individual was (Figure 2A) concentrated, with a median value of 14.33. Based on this, lung adenocarcinoma patients were then divided into AURKB^high^ and AURKB^low^ groups, with 245 samples each group. As shown in Figure 2B, 2C, higher expression of AURKB contributed to significantly worse overall survival (OS, p=0.002) and progression-free survival (PFS, p=0.013), indicating that AURKB is associated with poor prognosis in patients with lung adenocarcinoma. The data of multivariate cox analysis also showed that AURKB and N staging could be two independent prognostic factors of LUAD (HR, 95%CI: 1.672, 1.045-2.674 for AURKB, 1.897, 1.13-3.184 for N staging, Figure 2D), irrespective of T stage, M stage, smoking history and EGFR mutation status. The outcome data of 21 data sets involving 2912 patients with LUAD further confirmed that AURKB could be a prognostic factor in LUAD (HR, 95%CI: 1.22, 1.13-1.32, Figure 2E).

To evaluate the potential of AURKB as a prognostic marker in blood samples, we obtained the data of 80 LUAD tumor samples and 80 normal control samples from the GEO database (GSE20189). The original data of the chip were downloaded, and the R software package Affy was used to perform data conversion and RMA standardization for obtaining a gene expression profile data set. The expression of AURKB in tumor samples was found to be higher than that of control samples with significant margins (p=0.072, Supplementary Figure 1A). Further analysis of the changes in the expression of AURKB at the early and advanced stages of LUAD showed that AURKB was significantly high-expressed in tumors more advanced after Stage II (p=0.043, Supplementary Figure 1B). Angiogenic factors are key markers of tumor progression. The ssGSEA method was used to evaluate the scores of angiogenic factors in the samples, and the data indicated that the scores of angiogenic factors in tumor samples were significantly higher than in normal samples (p=0.00012, Supplementary Figure 1C). In LUAD patients, the expression of AURKB was greatly positively correlated with the angiogenic factor score (r=0.52, p<0.001, Supplementary Figure 1D). The above results indicated that the expression of AURKB was of a certain consistency in blood samples and tumor tissue samples.

### Genome-wide mRNA profiles associated with AURKB expression

**Figure 3:**
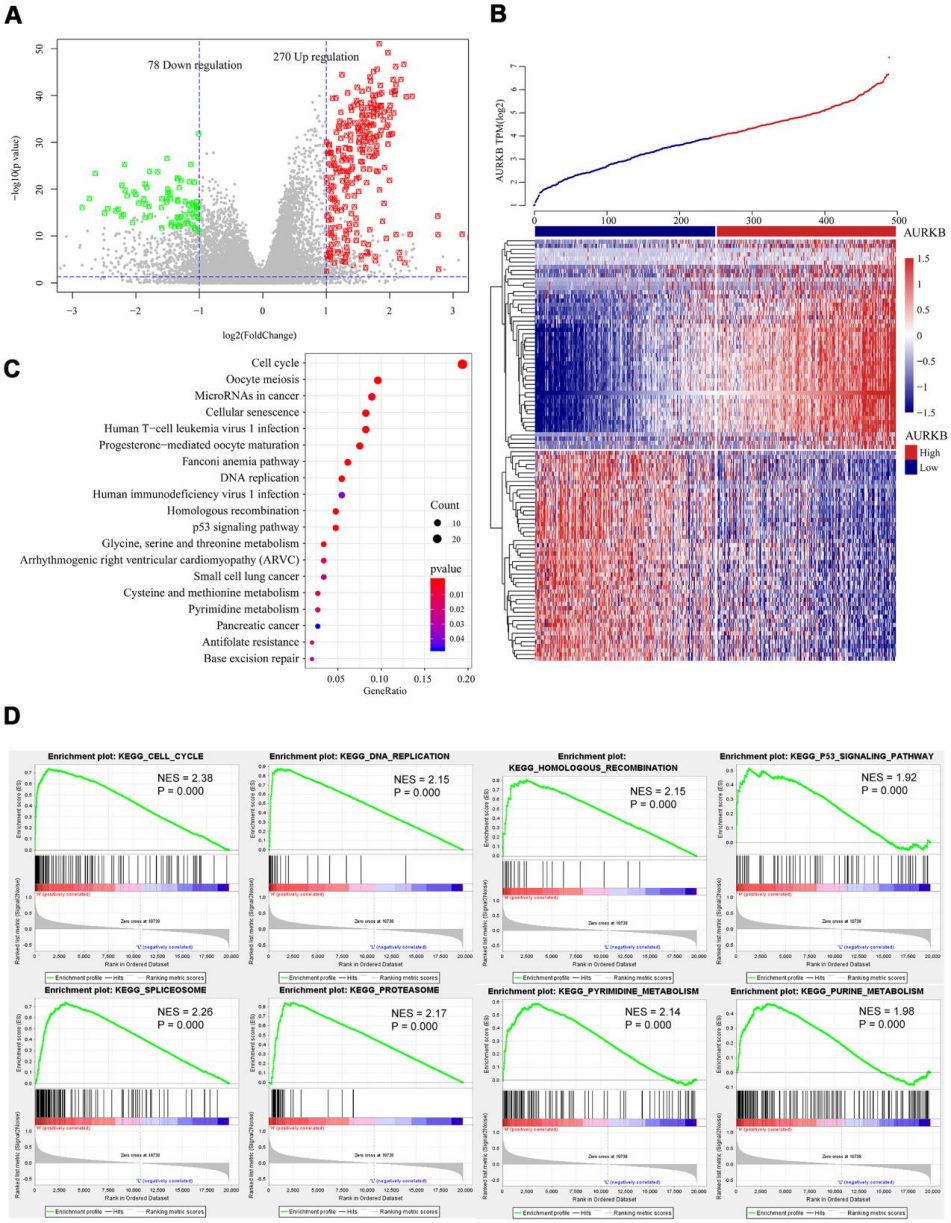
**Genome-wide mRNA profiles associated with AURKB expression.** (**A**, **B**) Volcano plot and heatmap showed the distribution and cluster of DEGs between AURKB^high^ and AURKB^low^ patients. (**C**) Top 20 pathways were identified through KEGG enrichment analysis by ClusterProfiler R-package, including cell cycle, DNA replication, homologous recombination, and p53 signaling pathway, etc. (**D**) Significant pathways enriched by GSEA analysis of DEGs between AURKB^high^ and AURKB^low^ patients were shown here: cell cycle, DNA replication, homologous recombination, p53 signaling pathway, spliceosome, proteasome, pyrimidine metabolism, and purine metabolism. Positive values indicate a higher correlation with AURKB^high^ patients, while negative values indicate association with AURKB^low^ patients.

To identify the key genes involved in the AURKB-mediated poor survival of LUAD and related biological functions or pathways, we compared the expressions of differentially expressed genes (DEGs) between AURKB^high^ group and AURKB^low^ group and examined their correlations with AURKB using the T-test and the rank-sum test, respectively (mRNAs were excluded if over 50% values are empty or zero). Only genes with correlation coefficients larger than 0.3, fold change >2, and the p-value < 0.01 either in the T-test or the rank-sum test were considered as DEGs. As visualized by volcano plot and heatmap (Figure 3A, 3B), 348 DEGs incorporating 270 upregulated genes and 78 downregulated genes were found. The top 10 upregulated and down-regulated genes identified by fold changes were listed in Table 2. The most correlated mRNA with AURKB was FAM64A, which is a marker of cell proliferation, and has been found to be associated with the aggressive growth of pancreatic cancer and breast cancer [16, 17]. Moreover, study indicated that FAM64A may also regulate Th17 cells and promote inflammation-associated cancers [18]. PRAME, an antigen high-expressed in various types of cancers, is involved in cell apoptosis, differentiation, proliferation, and metastasis [19], and it had the greatest fold change in the two groups. CDCA3 is the most significantly differentiated mRNA, and is already proven to be a prognostic factor in NSCLC patients [20]. Additionally, we further used ClusterProfiler [21] R-package to run KEGG enrichment analysis, and found that DEGs were enriched in 19 pathways (p<0.05), especially in cancer-related pathways including cell cycle, DNA replication, homologous recombination, and p53 signaling pathway (Figure 3C). These pathways were additionally validated by KEGG pathway enrichment analysis using GSEA. Meanwhile, we found some other pathways (24 pathways in total), such as purine metabolism, pyrimidine metabolism, spliceosome, and proteasome, were related to high expression of AURKB (Figure 3D). Since AURKB could mediate EGFR TKI resistance as above-mentioned, we also analyzed the relationship between 19 pathways and EGFR pathway by calculating the score of each patient in these pathways using ssGSEA. The results demonstrated that most of the 19 pathways were significant correlated with the EGFR pathway (89.5%) (Supplementary Figure 2A). However, for the AURKB^low^ group, no pathways met our criteria that both p-value and FDR q-value should be less than 0.05. Aberrant DEGs and enriched pathways identified in this study were closely associated with cancer formation and therapy resistance, which was consistent with our previous finding that AURKB mediated poor outcome for LUAD patients and might explain for the internal mechanism of action.

**Table 2:** Top 20 differentiated PCGs.

	**T test p value**	**Wilcox test p value**	**Fold Change**	**Spearman Correlation (R)**
PRAME	3.16E-06	6.65E-10	20.699	0.362
IGF2BP1	9.40E-08	3.79E-17	12.741	0.431
C12orf56	2.29E-10	1.67E-12	9.631	0.334
IGF2BP3	4.23E-11	1.01E-20	8.824	0.418
GFY	0.0012	1.61E-15	6.832	0.402
ANKRD18B	5.07E-11	1.20E-17	6.785	0.385
YBX2	5.62E-15	2.18E-18	6.765	0.477
MYBL2	1.43E-40	2.85E-63	5.116	0.713
ZNF695	4.60E-11	2.57E-26	4.998	0.584
UBE2C	1.88E-40	4.91E-62	4.796	0.639
CYP4B1	7.88E-17	1.53E-30	0.139	-0.318
ADH1B	9.95E-19	4.38E-33	0.150	-0.336
C16orf89	4.16E-24	5.64E-35	0.160	-0.384
SCTR	1.24E-15	4.92E-25	0.178	-0.317
SUSD2	1.50E-16	1.07E-29	0.184	-0.304
CD1E	4.17E-16	7.37E-29	0.201	-0.312
CACNA2D2	1.84E-16	3.60E-24	0.206	-0.304
SFTPD	5.84E-15	1.23E-21	0.213	-0.306
C1QTNF7	1.40E-21	6.06E-42	0.215	-0.377
ADAMTS8	2.94E-15	6.43E-30	0.218	-0.308

### Connections between microRNAs and AURKB

**Figure 4:**
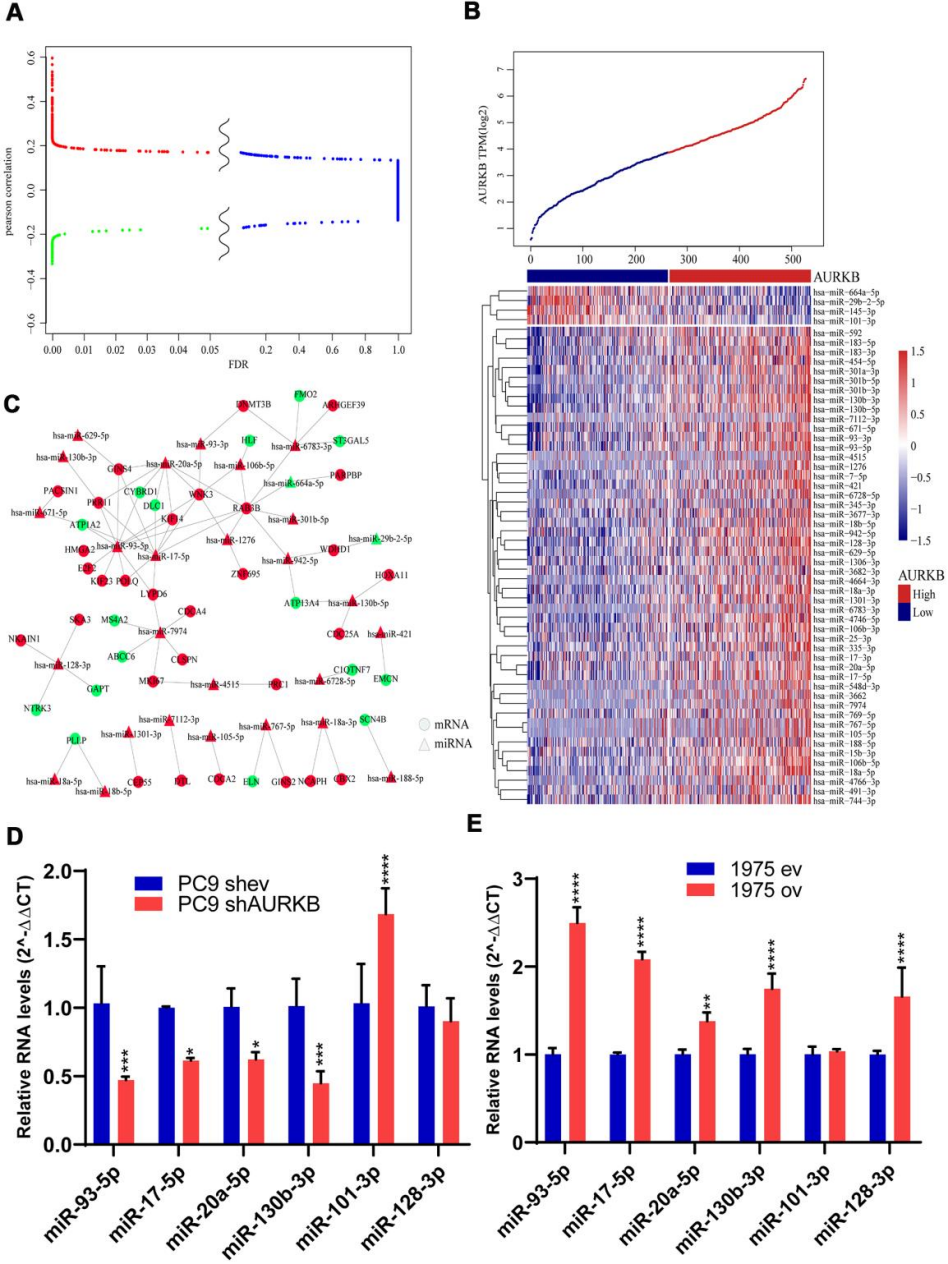
**MiRNAs associated with AURKB.** (**A**) Differential volcano map showed the distribution of microRNAs. Red color indicates microRNAs positively correlated with AURKB, green indicates negative ones (Pearson correlation, R>0.13 or < -0.13), and blue color indicates irrelevant microRNAs (FDR > 0.05). (**B**) Clusters of 54 identified miRNAs significantly associated with AURKB, and samples were arranged with increasing expression of AURKB from left to right. (**C**) Regulatory network analysis of significantly correlated mRNAs and microRNAs, red represents upregulation, and green represents downregulation. (**D**, **E**) Relative expression levels of miR-93-5p, miR-17-5p, miR-20a-5p, miR-130b-5p, miR-101-3p, and miR-128-3p in PC9 shev and PC9 shAURKB (AURKB knockdown) cells (**D**) and 1975 ev and 1975 ov (AURKB overexpression) cells (**E**) constructed using lentivirus, U6 served as a internal control (2^-ΔΔCT^ method, p<0.05, *; p<0.01, **; p<0.001, ***; p<0.0001, ****).

We also found certain DEGs were enriched in the miRNA pathways of LUAD, thus, genome-wide miRNA profile analysis was performed. In a further Pearson correlation analysis with AURKB, 926 miRNAs with at least 30% sample expression values were included. A total of 54 miRNAs met the threshold, with a FDR of less than 0.05 and a correlation coefficient of above 0.3. Specifically, 50 of these miRNAs were positively correlated with AURKB expression, while 4 were negatively correlated with AURKB expression. The distribution relationship of correlation coefficients with FDR is shown in Figure 4A. As displayed by the clustered heatmap (Figure 4B), only a small number of miRNAs were negatively correlated with AURKB, while most of the miRNAs were positively correlated with AURKB. Among them, the top miRNA with the highest positive correlation coefficient was miR-130b-3p. Previous research has been reported that miR-130b-3p present in exosomes contributes to CXCL12/CXCR4-induced colorectal cancer metastasis into liver [22] and downregulate PIEZO2, thereby activating the Hedgehog signaling pathway and leading to worsening breast cancer prognosis [23]. Moreover, miR-421 is previously identified as a prognostic biomarker indicating promoted tumor progression of non-small cell lung cancer (NSCLC) [24]. miR-29b-2-5p and miR-101-3p were negatively correlated with AURKB. From reviewing previous studies, miR-29b-2-5p is reported to target CBI-b to inhibit the proliferation of pancreatic cancer cells [25], and miR-101-3p could hinder the growth and metastasis of NSCLC through attenuating MALAT-1 mediated PI3K/AKT signal pathway [26].

Correlated miRNAs were analyzed by the T-test and rank-sum test to compare the expression changes between AURKB^high^ and AURKB^low^ groups. 49 miRNAs were screened based on FDR < 0.05 and fold change > 1.5. Then mirWallk database was used to predict the target genes of these differential miRNAs and to construct mRNA-miRNA regulatory network, as shown in Figure 4C. Further analysis of the relationship between miRNA in the network and EGFR pathway showed that 82% of miRNAs predicted were significantly correlated with EGFR pathway (Supplementary Figure 2B). Based on the scores calculated by the degree algorithm, the following top 10 hub genes were screened: miR-93-5p, RAB3B, miR-17-5p, miR-20a-5p, WNK3, miR-7974, miR-6783-3p, PRR11, GINS4 and miR-128-3p (Table 3). miR-93-5p, which was reported to promote the proliferation of NSCLC cells and is indicative of a poor prognosis, was found to enhance the transcription of several oncogenic genes, such as HMGA2, E2F2, KIF23, POLQ, PRR11, GINS4, WNK3, LYPD6, KIF14, RAB3B and downregulate the expressions of tumor suppressors such as ATP1A2 and CYBRD1. Taken together, these observations provided rational evidence for the prognostic role of AURKB in lung adenocarcinoma.

**Table 3:** Top 10 scores analyzed by degree algorithm.

**Name**	**Score**
hsa-miR-93-5p	12
RAB3B	9
hsa-miR-17-5p	9
hsa-miR-20a-5p	7
WNK3	6
hsa-miR-7974	6
hsa-miR-6783-3p	5
PRR11	4
GINS4	4
hsa-miR-128-3p	4

To further validate the association of identified miRNAs with AURKB *in vitro*, we tested the protein expression levels of AURKB among 4 lung adenocarcinoma cell lines, including A549, H1299, PC9 and H1975 (Supplementary Figure 3A). Lentiviral shRNA-mediated ARUKB inhibition in AURKB high-expressed PC9 cells and lentiviral AURKB-mediated overexpression in AURKB low-expressed H1975 cells were confirmed by western blotting and quantitative PCR (Supplementary Figure 3B, 3C). Knockdown of AURKB in PC9 cells significantly downregulated the expressions of miR-93-5p, miR-17-5p, miR-20a-5p, miR-130b-3p and upregulated the expressions of miR-101-3p (Figure 4D). While overexpression of AURKB in H1975 cells significantly upregulated the expressions of miR-93-5p, miR-17-5p, miR-20a-5p, miR-130b-3p, and miR-128-3p (Figure 4E).

### Whole-genome methylation profiles associated with AURKB

**Figure 5:**
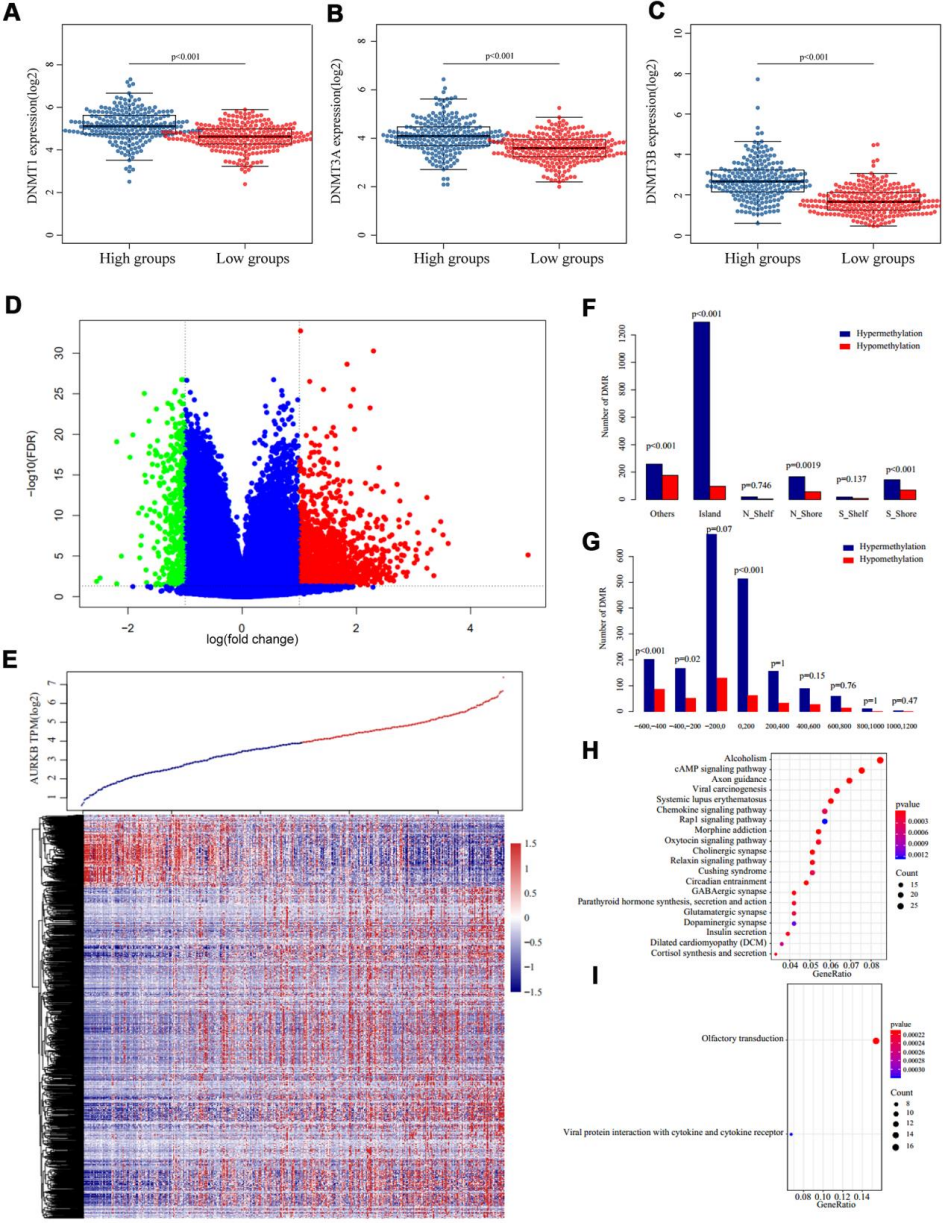
**Methylation patterns associated with AURKB expression.** (**A**–**C**) Expression of three DNA methyltransferases ((**A**) DNMT1, (**B**) DNMT3A, (**C**) DNMT3B) in AURKB^high^ and AURKB^low^ groups, p value as indicated in the figure. (**D**, **E**) Volcano plot (**D**) and heatmap (**E**) of differentially methylated regions between AURKB^high^ and AURKB^low^ groups (red and green mean significantly DMRs, cutoff fold change 1.3, FDR 0.05). (**F**, **G**) Distribution of DMRs on gene’s different structural regions and the distance to TSS. (**H**, **I**) KEGG analysis of hypermethylated genes were enriched into 36 pathways, top 20 were listed in (**H**), while hypomethylated genes were only enriched into two pathways, as shown in (**I**).

DNA methylation is an important epigenetic mechanism regulating gene expressions through three DNA methyltransferases (DNMT1, DNMT3A, and DNMT3B) and affects cancer cell behaviors. We analyzed differences in the transcription of the three methyltransferases, and as shown in Figure 5A–5C, expressions of the three were significantly higher in AURKB^high^ than AURKB^low^ groups. 476 samples with methylation profiles were included to further examine the methylation differences between the AURKB^high^ and AURKB^low^ groups. Methylation sites were excluded if more than 30% of the samples showed no values or happened to be the cross-reactive CpG sites in the Illumina Infinium Human Methylation 450 microarray. After filling up missing values using the KNN method in the R package ‘impute’, a total of 195,622 methylation sites were obtained and then compared with the R package ‘limma’, according to the thresholds of fold change > 1.3 and FDR < 0.05. The volcano map in Figure 5D showed that the hypermethylation sites were significantly more than hypomethylation sites (1900 and 411, respectively). Heatmap of these differential methylation sites is shown in Figure 5E.

By examining the position distribution of the 2311 differential methylation regions (DMR) around the CpG islands, as shown in Figure 5F, all the positions had more hypermethylated DMRs, although the differences were not significant in N_Shelf or S-Shelf (Island: P<0.001, N_Shore: P=0.0019, S_Shore and Others: P<0.001). As expected, most hypermethylated DMRs centered on the CpG islands. Finally, we determined the distance of methylation sites to the transcription starting site (TSS), as presented in Figure 5G, most hypomethylated DMRs and hypermethylated DMRs were on the upstream of TSS (-600 – +200), and hypermethylated DMRs fell to -200 – +200 regions. Generally, hypermethylated sites tended to distribute around TSS, while hypomethylated sites tended to distribute on the upstream of TSS.

We extracted the corresponding genes of these DMRs based on the TSS and ran a KEGG enrichment analysis using R package ClusterProfiler. The data demonstrated that genes corresponding to hypermethylation were enriched into 36 pathways, such as cAMP signaling pathway, axon guidance, viral carcinogenesis, and chemokine signaling pathway (p<0.05, Figure 5H). However, the relative hypomethylated genes were only enriched into two pathways, which were olfactory transduction and viral protein in interaction with cytokine and cytokine receptors (Figure 5I).

### Tumor suppressors DLC1 and HLF negatively correlated with ARUKB and its associated miRNAs and methylation modification

**Figure 6:**
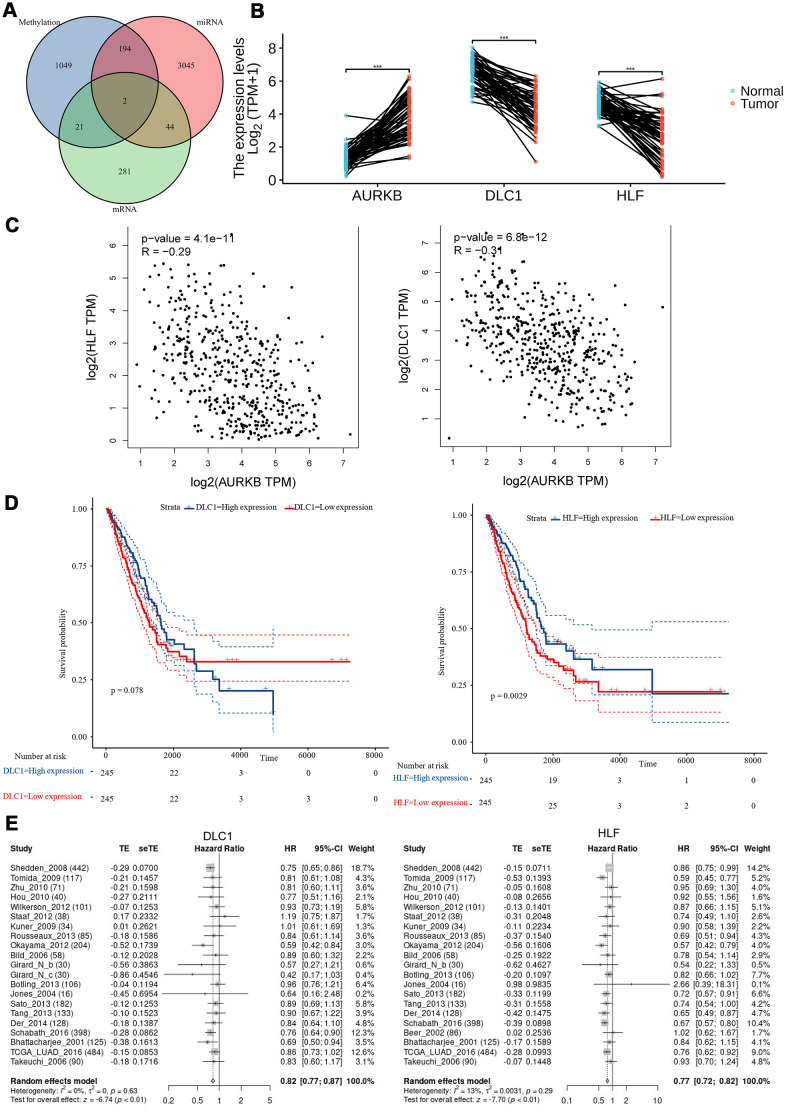
**Tumor suppressors (DLC1 and HLF) negatively correlated with AURKB and its associated miRNAs and methylation modification.** (**A**) Venn plot of identified DEGs, differentially expressed microRNAs, and DMRs; (**B**) the expression levels (Log_2_(TPM+1)) of AURKB, DLC1, and HLF in paired tumor and adjacent tissues in the TCGA-LUAD cohort. (**C**) The correlation of HLF or DLC1 with AURKB were analyzed by GEPIA website (HLF: R = -0.29, p = 4.1e-11; DLC1: R = -0.31, p = 5.8e-12). (**D**) Low expression of DLC1 or HLF led to poor overall survival in the TCGA-LUAD cohort (p = 0.078, 0.0029, respectively). (**E**) Meta-analysis of DLC1 or HLF expression in the prediction of overall survival in lung adenocarcinoma patients from different datasets by lung cancer explorer (LCE).

As we have obtained the genome-wide mRNAs, miRNA-regulated genes, and methylation-regulated genes profiles, we then went on to investigate whether these three profiles shared genes in common that may have close crosstalk with AURKB to promote LUAD aggressiveness. As shown in Figure 6A, two genes, DLC1 and HLF, were present in all the three profiles. The differential mRNA expression levels of both genes were further confirmed using paired lung adenocarcinoma tumor and adjacent tissues in TCGA (p<0.001 for both genes, Figure 6B). Gene correlation analysis using GEPIA web-tool (http://gepia.cancer-pku.cn/) also confirmed that AURKB was significantly negatively correlated with DLC1 and HLF (R= -0.29, -0.34, respectively, Figure 6C). Survival analysis of included LUAD patients from the TCGA database also showed an apparent difference between HLF^high^ and HLF^low^ patients (p=0.0029), and DLC1 was determined to be a protective factor to the survival of patients with LUAD (p=0.078) (divided by the median value, Figure 6D). LCE online survival analysis demonstrated that low expressions of DLC1 and HLF were associated with a shorter survival time of LUAD patients (HR[95%CI], 0.82[0.77; 0.87], 0.77[0.72;0.82], respectively, Figure 6E).

## DISCUSSION

Abnormal proliferation of cancer cells play an essential role in both cancer formation and metastasis [27]. Cancer cells will consume nutrients and reprogram energy metabolism of normal cells, including stromal cells and immune cells, thereby further enhancing cancer survival and aggressive growth [28]. Cell mitosis restricts the fast proliferation of cancer cells, but most cancer cells overexpress mitosis-related proteins such as AURK family member AURKA and AURKB to affect chromosome segregation and cell cycle and cause unequal distribution of genome, producing aneuploid cells in cancers [29]. A retrospective study with a cohort of 132 NSCLC patients confirmed that AURKA could be an independent prognostic factor (HR[95%CI]: 1.81[1.16-2.84]) [30]. Here we identified AURKB as an independent prognostic factor in LUAD patients using the TCGA-LUAD cohort and further confirmed our findings through multiple datasets in LCE.

EGFR wild type and mutated types tend to have a similar expression level of AURKB, although they showed different survival outcomes by application of EGFR-TKIs. However, as mentioned above, the majority of AURKB associated pathways and miRNAs were significantly correlated with EGFR signaling pathway, and AURKB has been reported to be responsible for the therapy resistance to EGFR-TKIs in patients without secondary resistant mutations during progression, suggesting that AURKB could be a vital factor as a downstream effector of EGFR pathway contributing to tumor progression.

We further explore the mechanisms and essential biofunctions or pathways of AURKB in LUAD from the aspects of gene expression, miRNA expression, and methylation profiles. As previously demonstrated, AURKB was correlated with FAM64A, PRAME, CDCA3, etc., which are all highly related to cancers. DEGs upregulated in AURKB^high^ patients were enriched in the cell cycle, DNA replication, homologous recombination, and p53 signaling pathway, which confirmed the role of AURKB in the mitosis. Moreover, we also found that some AURKB-associated genes were also involved in purine metabolism, pyrimidine metabolism, spliceosome, and proteasome pathways, indicating that the fundamental processes of a fast proliferation of LUAD cells were also regulated by AURKB either through nucleotide metabolism, mRNA maturation or posttranslational protein modifications.

For miRNA profiles, in this study, most miRNAs were positively correlated with AURKB expression, which is consistent with enriched pathway ‘miRNAs in cancer’ and indicated that AURKB may also regulate the genome-wide changes to facilitate LUAD growth through miRNAs. miR-93-5p, a hub miRNA in the profile, was predicted to activate RAB3B, WNK3, GINS4, PRR11 hub genes. Specifically, RAB3B is a member of the ras oncogene family currently remain largely unexplored; WNK3 is a serine-threonine protein kinase functioning as a positive regulator of the transcellular Ca2+ transport pathway and increases cell survival in a caspase 3-dependent pathway [31]. GINS4 was reported to contribute to the poor outcome in lung adenocarcinomas [32]. Recent research found that PRR11 could facilitate F-actin polymerization and disrupt the F-actin cytoskeleton, thereby causing aberrant nuclear lamina assembly and chromatin reorganization in NSCLC [33]. To the best of our knowledge, previous researches have shown many cases of such miRNA-mRNA pairs [34], but the modulation of those genes by miR-93-5p has not been reported yet.

Other epigenetic modifications and methylation changes were also affected by AURKB, as we demonstrated that DNA methyltransferases were remarkably increased in AURKB^high^ patients. In our study, hypermethylation, which reflects transcription repression functions, mostly occured within CpG island around TSS [35]. It was possible that those abnormal methylations might accompany the adverse prognosis. Interestingly, in this research, two tumor suppressors (DLC1 and HLF) were both found to be closely correlated with AURKB and AURKB-associated differentially expressed miRNAs and hypermethylation. DLC1 is a tumor suppressor in many different cancer types, including in lung cancer [36, 37] and could inhibit cancer cells through Rho GTPase accelerating proteins (GAP) dependent- and independent-mechanisms [38]. HLF, which is high-expressed in liver tissues but low-expressed lung tissues, is initially identified as a protein related to TCF3 driving acute lymphoblastic leukemia [39], but it is also demonstrated to be robustly hypermethylated in NSCLC when compared to normal tissues [40, 41]. These two genes were closely related to AURKB through methylation modification and miRNA regulation, however, this remained to be validated by our team.

Smoking is a high risk factor and driving event for lung cancer, as we found here, significantly correlated with higher expression of AURKB in LUAD. To analyze the effect of smoking history on AURKB expression, we compared the profiles of DEGs, differentially expressed miRNAs, and methylation differences between AURKB^high^ and AURKB^low^ groups in smokers and non-smokers separately, according to the thresholds of a two-fold difference and p <0.01. Generally, at different omics levels, the differences between AURKB^high^ and AURKB^low^ groups in patients with smoking history were greater than that in the non-smoking group (Supplementary Figure 4). Interestingly, >80% varied molecular features in the non-smoking group were commonly found in the smoking group, suggesting that smoking might be a main factor affecting AURKB expression and altering LUAD patient’s genome characteristics.

In summary, this study found that AURKB may be an effective factor to predict LUAD patients’ survival. Also, personalized drugs with AURKB could be designed to treat patients with AURKB overexpression and to overcome acquired resistance to chemo reagents or targeting agents. Recently, a phase I dose-escalation study showed that Chiauranib could simultaneously inhibits angiogenesis-related kinases, AURKB, and chronic inflammation-related kinase CSF-1R and is tested to be tolerable even in advanced solid tumors, especially in NSCLC (4 in 5 patients with disease control) [42]. However, AURKB inhibitors were largely unavailable in clinical practice, which requires further development of effective AURKB inhibitors. Moreover, the intrinsic regulatory mechanism involving gene expression, miRNAs and methylation through which AURKB could be better used as a potential and safe target should be deeply studied.

## MATERIALS AND METHODS

### Data acquisition and preprocessing

The expression profiles of mRNAs and miRNAs were sequenced by RNA-seq and 450K genome-wide methylation profile. LUAD clinical data, including 513 tumor samples and 59 para-cancer tissues in the Cancer Genome Atlas (TCGA) were downloaded from Genomic Data Commons Data Portal (portal.gdc.cancer.gov, 05/23/19). According to the clinical data, samples acquired from patients with follow-up time more than 30 days were selected to match the expression profiles of mRNAs, miRNAs, and methylation information for final analyses. The detailed statistical description is listed in Table 4. The cancer samples were subsequently divided into AURKB^high^ expression and AURKB^low^ expression subgroups based on the median value and were used for further analyses. Lung Cancer Explorer (LCE, http://lce.biohpc.swmed.edu/lungcancer/) online analysis was used to compare the expression levels between tumor and normal tissues and survival changes based on AURKB expressions. All the results were calculated by the meta-analysis of multiple LUAD datasets from TCGA and Gene Expression Omnibus (GEO).

**Table 4:** Data and sample counts.

**Data type**	**Samples**	**Total**
miRNA	529	2155
PCG	490	19754
Methylation	476	195622

### Cell lines

LUAD cell lines (A549, H1299, H1975, and PC9) were purchased from the American Type Culture Collection (ATCC) Cell Bank and cultured in RPMI 1640 (Gibco, catalog: 31800-022) at 37° C in a 5% CO_2_ atmosphere. All the media were supplemented with 10% fetal bovine serum (serana, catalog: S-FBS-EU-015), 100 U/ml penicillin and 100 μg/ml streptomycin.

### Quantitative RT-PCR

Total RNA from cell pellets was extracted using the Total RNA Miniprep Kit (AxyPrep, USA) according to the manufacturer’s protocol. The quantity and quality of extracted RNA were assessed with a Nanodrop 2000c (Thermo Scientific, USA). Then, complementary DNA was synthesized from 500 ng of total RNA using PrimeScript™ RT Master Mix kit (Takara, Japan). For the stem-loop reverse transcription of microRNAs, PrimeScript™ RT Reagent Kit (Takara, Japan) was used to syntheze cDNA with miRNA-unique stem-loop primers. PCR analysis was performed in a Applied Biosystems 7500 Fast Real-Time PCR System (Foster City, USA) with SYBR Premix Ex Taq II (Takara, Japan). The expressions of miRNAs and mRNAs were calculated using the 2^-ΔΔCT^ method, with U6 and ACTB served as normalization controls, respectively. All primers used are listed in Table 5.

**Table 5:** Primers for the quantative RT-PCR.

**SYMBOL**	**Forward Primer**	**Reverse Primer (mRNA) / Stem-loop RT Primer (microRNA)**
AURKB	CGCAGAGAGATCGAAATCCAG	AGATCCTCCTCCGGTCATAAAA
ACTB	CATGTACGTTGCTATCCAGGC	CTCCTTAATGTCACGCACGAT
U6	CTCGCTTCGGCAGCACA	AACGCTTCACGAATTTGCGT
hsa-miR-93-5p	AACGGCCAAAGTGCTGTTCG	RT:GTCGTATCCAGTGCAGGGTCCGAGGTATTCGCACTGGATACGACCTACCTG
hsa-miR-17-5p	AGCGAGGCCAAAGTGCTTACAG	RT:GTCGTATCCAGTGCAGGGTCCGAGGTATTCGCACTGGATACGACCTACCTG
hsa-miR-20a-5p	AGGCGTGCTAAAGTGCTTATAG	RT:GTCGTATCCAGTGCAGGGTCCGAGGTATTCGCACTGGATACGACCTACCTG
hsa-miR-130b-3p	AAGCGACCCAGTGCAATGATG	RT:GTCGTATCCAGTGCAGGGTCCGAGGTATTCGCACTGGATACGACATGCCCT
hsa-miR-101-3p	AAGCGACCTACAGTACTGTGA	RT:GTCGTATCCAGTGCAGGGTCCGAGGTATTCGCACTGGATACGACTTCAGT
hsa-miR-128-3p	AACAGTGTCACAGTGAACCG	RT:GTCGTATCCAGTGCAGGGTCCGAGGTATTCGCACTGGATACGACAAAGAG
miR universal R		GTCGTATCCAGTGCAGGGTCC

### Western blot

Total protein were extracted from cell pellets using RIPA buffer. Protein concentration was determined by BCA assay (Pierce, Rockford, IL, USA) using bovine serum albumin as a standard. 30 ug protein samples were separated in 10% SDS-PAGE gel and transferred to a PVDF membrane, which was blotted with antibodies (primary antibodies rabbit anti-AURKB (Cat# ET1610-25, 1:1000), rabbit anti-GAPDH (Cat# ER1706-83, 1:3000), rabbit anti-beta-actin (Cat#R1207-1, 1:3000)), then rinsed and incubated with secondary IgG-HRP antibodies goat anti-rabbit (Cat# HA1019, 1:3000) and goat anti-mouse (Cat#HA1020, 1:3000) according to the manufacturer’s instructions. All antibodies came from HuaBio in China.

### AURKB overexpression or inhibition in lung adenocarcinoma cells

Briefly, pLenti-CMV-GFP-Puro-AURKB and pPLK/GFP+Puro-AURKB shRNA were constructed on the backbone of pLenti-CMV-GFP-Puro and pPLK/GFP+Puro plasmids, respectively, and each was then co-tranfected with lentiviral packaging plasmids pMD2.G and psPAX2 (Addgene, USA) into HEK 293T cells to generate lentivirus particles, followed by the transfection of the particles into target cells. The cells were incubated with lentivirus for 24 hours (h) and then replaced with fresh medium. When cells stably grew to proliferative status, cell pellets were collected for protein and total RNA extraction.

### Statistical analysis

The chi-squared test was used to examine the correlation between AURKB expression and clinical or pathological variables. The endpoint event overall survival (OS) was defined as the time starting from the occurrence of LUAD to death due to any cause. Progression-free survival (PFS) was defined as the time starting from the onset of LUAD to progression after the first-line treatment or death. Kaplan-Meier analysis with the log-rank test was applied to compare the survival differences between two groups of patients, while multivariate cox regression analysis was used to examine potential independent factors related to survival. Student’s t-test and false discovery rate (FDR) were used to identify differences in mRNAs, miRNAs, and methylation data between AURKB^high^ and AURKB ^low^ expression groups. All the analyses were performed using the R 3.6 software packages.

## Supplementary Material

Supplementary Figures

## References

[1] Solassol I, Pinguet F, Quantin X. FDA- and EMA-approved tyrosine kinase inhibitors in advanced EGFR-mutated non-small cell lung cancer: safety, tolerability, plasma concentration monitoring, and management. Biomolecules. 2019; 9:668. 10.3390/biom9110668 31671561 PMC6921037

[2] Proto C, Ferrara R, Signorelli D, Lo Russo G, Galli G, Imbimbo M, Prelaj A, Zilembo N, Ganzinelli M, Pallavicini LM, De Simone I, Colombo MP, Sica A, et al. Choosing wisely first line immunotherapy in non-small cell lung cancer (NSCLC): what to add and what to leave out. Cancer Treat Rev. 2019; 75:39–51. 10.1016/j.ctrv.2019.03.004 30954906

[3] den Hollander J, Rimpi S, Doherty JR, Rudelius M, Buck A, Hoellein A, Kremer M, Graf N, Scheerer M, Hall MA, Goga A, von Bubnoff N, Duyster J, et al. Aurora kinases A and B are up-regulated by Myc and are essential for maintenance of the malignant state. Blood. 2010; 116:1498–505. 10.1182/blood-2009-11-251074 20519624 PMC2938839

[4] Hartsink-Segers SA, Zwaan CM, Exalto C, Luijendijk MW, Calvert VS, Petricoin EF, Evans WE, Reinhardt D, de Haas V, Hedtjärn M, Hansen BR, Koch T, Caron HN, et al. Aurora kinases in childhood acute leukemia: the promise of aurora B as therapeutic target. Leukemia. 2013; 27:560–68. 10.1038/leu.2012.256 22940834 PMC3593181

[5] Enjoji M, Iida S, Sugita H, Ishikawa T, Uetake H, Inokuchi M, Yamada H, Kojima K, Sugihara K. BubR1 and AURKB overexpression are associated with a favorable prognosis in gastric cancer. Mol Med Rep. 2009; 2:589–96. 10.3892/mmr_00000142 21475871

[6] Nie M, Wang Y, Yu Z, Li X, Deng Y, Wang Y, Yang D, Li Q, Zeng X, Ju J, Liu M, Zhao Q. AURKB promotes gastric cancer progression via activation of CCND1 expression. Aging (Albany NY). 2020; 12:1304–21. 10.18632/aging.102684 31982864 PMC7053608

[7] Morozova O, Vojvodic M, Grinshtein N, Hansford LM, Blakely KM, Maslova A, Hirst M, Cezard T, Morin RD, Moore R, Smith KM, Miller F, Taylor P, et al. System-level analysis of neuroblastoma tumor-initiating cells implicates AURKB as a novel drug target for neuroblastoma. Clin Cancer Res. 2010; 16:4572–82. 10.1158/1078-0432.CCR-10-0627 20651058

[8] Burgess EF, Livasy C, Trufan SJ, Hartman A, Guerrieri R, Naso C, Clark PE, Grigg C, Symanowski JT, Raghavan D. Impact of Aurora kinase A and B expression on response to neoadjuvant chemotherapy and patient outcome in muscle-invasive bladder cancer (MIBC). J Clinic Oncol. 2019; 37 (Suppl 7):393. 10.1200/JCO.2019.37.7_suppl.393 31597600

[9] Wan B, Huang Y, Liu B, Lu L, Lv C. AURKB: a promising biomarker in clear cell renal cell carcinoma. PeerJ. 2019; 7:e7718. 10.7717/peerj.7718 31576249 PMC6752188

[10] Smith SL, Bowers NL, Betticher DC, Gautschi O, Ratschiller D, Hoban PR, Booton R, Santibáñez-Koref MF, Heighway J. Overexpression of aurora B kinase (AURKB) in primary non-small cell lung carcinoma is frequent, generally driven from one allele, and correlates with the level of genetic instability. Br J Cancer. 2005; 93:719–29. 10.1038/sj.bjc.6602779 16222316 PMC2361619

[11] Dos Santos EO, Carneiro-Lobo TC, Aoki MN, Levantini E, Bassères DS. Aurora kinase targeting in lung cancer reduces KRAS-induced transformation. Mol Cancer. 2016; 15:12. 10.1186/s12943-016-0494-6 26842935 PMC4739397

[12] Yu J, Zhou J, Xu F, Bai W, Zhang W. High expression of aurora-B is correlated with poor prognosis and drug resistance in non-small cell lung cancer. Int J Biol Markers. 2018; 33:215–21. 10.1177/1724600817753098 29707994

[13] Bertran-Alamillo J, Cattan V, Schoumacher M, Codony-Servat J, Giménez-Capitán A, Cantero F, Burbridge M, Rodríguez S, Teixidó C, Roman R, Castellví J, García-Román S, Codony-Servat C, et al. AURKB as a target in non-small cell lung cancer with acquired resistance to anti-EGFR therapy. Nat Commun. 2019; 10:1812. 10.1038/s41467-019-09734-5 31000705 PMC6472415

[14] Woo JK, Kang JH, Shin D, Park SH, Kang K, Nho CW, Seong JK, Lee SJ, Oh SH. Daurinol enhances the efficacy of radiotherapy in lung cancer via suppression of aurora kinase A/B expression. Mol Cancer Ther. 2015; 14:1693–704. 10.1158/1535-7163.MCT-14-0960 25882311

[15] Cai L, Lin S, Girard L, Zhou Y, Yang L, Ci B, Zhou Q, Luo D, Yao B, Tang H, Allen J, Huffman K, Gazdar A, et al. LCE: an open web portal to explore gene expression and clinical associations in lung cancer. Oncogene. 2019; 38:2551–64. 10.1038/s41388-018-0588-2 30532070 PMC6477796

[16] Zhang J, Qian L, Wu J, Lu D, Yuan H, Li W, Ying X, Hu S. Up-regulation of FAM64A promotes epithelial-to-mesenchymal transition and enhances stemness features in breast cancer cells. Biochem Biophys Res Commun. 2019; 513:472–78. 10.1016/j.bbrc.2019.03.207 30979502

[17] Jiao Y, Fu Z, Li Y, Zhang W, Liu Y. Aberrant FAM64A mRNA expression is an independent predictor of poor survival in pancreatic cancer. PLoS One. 2019; 14:e0211291. 10.1371/journal.pone.0211291 30695070 PMC6351057

[18] Xu ZS, Zhang HX, Li WW, Ran Y, Liu TT, Xiong MG, Li QL, Wang SY, Wu M, Shu HB, Xia H, Wang YY. FAM64A positively regulates STAT3 activity to promote Th17 differentiation and colitis-associated carcinogenesis. Proc Natl Acad Sci USA. 2019; 116:10447–52. 10.1073/pnas.1814336116 31061131 PMC6534998

[19] Xu Y, Zou R, Wang J, Wang ZW, Zhu X. The role of the cancer testis antigen PRAME in tumorigenesis and immunotherapy in human cancer. Cell Prolif. 2020; 53:e12770. 10.1111/cpr.12770 32022332 PMC7106952

[20] Adams MN, Burgess JT, He Y, Gately K, Snell C, Zhang SD, Hooper JD, Richard DJ, O’Byrne KJ. Expression of CDCA3 is a prognostic biomarker and potential therapeutic target in non-small cell lung cancer. J Thorac Oncol. 2017; 12:1071–84. 10.1016/j.jtho.2017.04.018 28487093

[21] Yu G, Wang LG, Han Y, He QY. clusterProfiler: an R package for comparing biological themes among gene clusters. OMICS. 2012; 16:284–87. 10.1089/omi.2011.0118 22455463 PMC3339379

[22] Wang D, Wang X, Si M, Yang J, Sun S, Wu H, Cui S, Qu X, Yu X. Exosome-encapsulated miRNAs contribute to CXCL12/CXCR4-induced liver metastasis of colorectal cancer by enhancing M2 polarization of macrophages. Cancer Lett. 2020; 474:36–52. 10.1016/j.canlet.2020.01.005 31931030

[23] Lou W, Liu J, Ding B, Jin L, Xu L, Li X, Chen J, Fan W. Five miRNAs-mediated PIEZO2 downregulation, accompanied with activation of hedgehog signaling pathway, predicts poor prognosis of breast cancer. Aging (Albany NY). 2019; 11:2628–52. 10.18632/aging.101934 31058608 PMC6535055

[24] Li Y, Cui X, Li Y, Zhang T, Li S. Upregulated expression of miR-421 is associated with poor prognosis in non-small-cell lung cancer. Cancer Manag Res. 2018; 10:2627–33. 10.2147/CMAR.S167432 30147363 PMC6095112

[25] Li C, Dong Q, Che X, Xu L, Li Z, Fan Y, Hou K, Wang S, Qu J, Xu L, Wen T, Yang X, Qu X, Liu Y. MicroRNA-29b-2-5p inhibits cell proliferation by directly targeting -b in pancreatic ductal adenocarcinoma. BMC Cancer. 2018; 18:681. 10.1186/s12885-018-4526-z 29940895 PMC6019739

[26] Zhang X, He X, Liu Y, Zhang H, Chen H, Guo S, Liang Y. MiR-101-3p inhibits the growth and metastasis of non-small cell lung cancer through blocking PI3K/AKT signal pathway by targeting MALAT-1. Biomed Pharmacother. 2017; 93:1065–73. 10.1016/j.biopha.2017.07.005 28738500

[27] Feitelson MA, Arzumanyan A, Kulathinal RJ, Blain SW, Holcombe RF, Mahajna J, Marino M, Martinez-Chantar ML, Nawroth R, Sanchez-Garcia I, Sharma D, Saxena NK, Singh N, et al. Sustained proliferation in cancer: mechanisms and novel therapeutic targets. Semin Cancer Biol. 2015; 35:S25–54. 10.1016/j.semcancer.2015.02.006 25892662 PMC4898971

[28] Ocaña MC, Martínez-Poveda B, Quesada AR, Medina MÁ. Metabolism within the tumor microenvironment and its implication on cancer progression: an ongoing therapeutic target. Med Res Rev. 2019; 39:70–113. 10.1002/med.21511 29785785

[29] Tang A, Gao K, Chu L, Zhang R, Yang J, Zheng J. Aurora kinases: novel therapy targets in cancers. Oncotarget. 2017; 8:23937–54. 10.18632/oncotarget.14893 28147341 PMC5410356

[30] Al-Khafaji AS, Marcus MW, Davies MP, Risk JM, Shaw RJ, Field JK, Liloglou T. AURKA mRNA expression is an independent predictor of poor prognosis in patients with non-small cell lung cancer. Oncol Lett. 2017; 13:4463–68. 10.3892/ol.2017.6012 28588715 PMC5452881

[31] Moniz S, Jordan P. Emerging roles for WNK kinases in cancer. Cell Mol Life Sci. 2010; 67:1265–76. 10.1007/s00018-010-0261-6 20094755 PMC11115774

[32] Yang R, Liu N, Chen L, Jiang Y, Shi Y, Mao C, Liu Y, Wang M, Lai W, Tang H, Gao M, Xiao D, Wang X, et al. LSH interacts with and stabilizes GINS4 transcript that promotes tumourigenesis in non-small cell lung cancer. J Exp Clin Cancer Res. 2019; 38:280. 10.1186/s13046-019-1276-y 31253190 PMC6599244

[33] Zhang L, Zhang Y, Lei Y, Wei Z, Li Y, Wang Y, Bu Y, Zhang C. Proline-rich 11 (PRR11) drives F-actin assembly by recruiting the actin-related protein 2/3 complex in human non-small cell lung carcinoma. J Biol Chem. 2020; 295:5335–49. 10.1074/jbc.RA119.012260 32169900 PMC7170533

[34] Stavast CJ, Erkeland SJ. The non-canonical aspects of MicroRNAs: many roads to gene regulation. Cells. 2019; 8:1465. 10.3390/cells8111465 31752361 PMC6912820

[35] Ando M, Saito Y, Xu G, Bui NQ, Medetgul-Ernar K, Pu M, Fisch K, Ren S, Sakai A, Fukusumi T, Liu C, Haft S, Pang J, et al. Chromatin dysregulation and DNA methylation at transcription start sites associated with transcriptional repression in cancers. Nat Commun. 2019; 10:2188. 10.1038/s41467-019-09937-w 31097695 PMC6522544

[36] Wang D, Qian X, Rajaram M, Durkin ME, Lowy DR. DLC1 is the principal biologically-relevant down-regulated DLC family member in several cancers. Oncotarget. 2016; 7:45144–57. 10.18632/oncotarget.9266 27174913 PMC5216712

[37] Popescu NC, Goodison S. Deleted in liver cancer-1 (DLC1): an emerging metastasis suppressor gene. Mol Diagn Ther. 2014; 18:293–302. 10.1007/s40291-014-0086-3 24519699 PMC4032595

[38] Du X, Qian X, Papageorge A, Schetter AJ, Vass WC, Liu X, Braverman R, Robles AI, Lowy DR. Functional interaction of tumor suppressor DLC1 and caveolin-1 in cancer cells. Cancer Res. 2012; 72:4405–16. 10.1158/0008-5472.CAN-12-0777 22693251 PMC6264793

[39] Inaba T, Roberts WM, Shapiro LH, Jolly KW, Raimondi SC, Smith SD, Look AT. Fusion of the leucine zipper gene HLF to the E2A gene in human acute B-lineage leukemia. Science. 1992; 257:531–34. 10.1126/science.1386162 1386162

[40] Lokk K, Vooder T, Kolde R, Välk K, Võsa U, Roosipuu R, Milani L, Fischer K, Koltsina M, Urgard E, Annilo T, Metspalu A, Tõnisson N. Methylation markers of early-stage non-small cell lung cancer. PLoS One. 2012; 7:e39813. 10.1371/journal.pone.0039813 22768131 PMC3387223

[41] Xu W, Lu J, Zhao Q, Wu J, Sun J, Han B, Zhao X, Kang Y. Genome-wide plasma cell-free DNA methylation profiling identifies potential biomarkers for lung cancer. Dis Markers. 2019; 2019:4108474. 10.1155/2019/4108474 30867848 PMC6379867

[42] Sun Y, Yang L, Hao X, Liu Y, Zhang J, Ning Z, Shi Y. Phase I dose-escalation study of chiauranib, a novel angiogenic, mitotic, and chronic inflammation inhibitor, in patients with advanced solid tumors. J Hematol Oncol. 2019; 12:9. 10.1186/s13045-018-0695-0 30642372 PMC6332596

