# Supplementary material for: Multi-omics analysis identifies potential mechanisms of AURKB in mediating poor outcome of lung adenocarcinoma
Source: Aging (Albany NY). 2021 Feb 17;13(4):5946–66. doi: 10.18632/aging.202517 (PMC7950220; doi:10.18632/aging.202517)
Supplement: Supplementary Figures [file aging-13-202517-s001.pdf]

SUPPLEMENTARY FIGURES

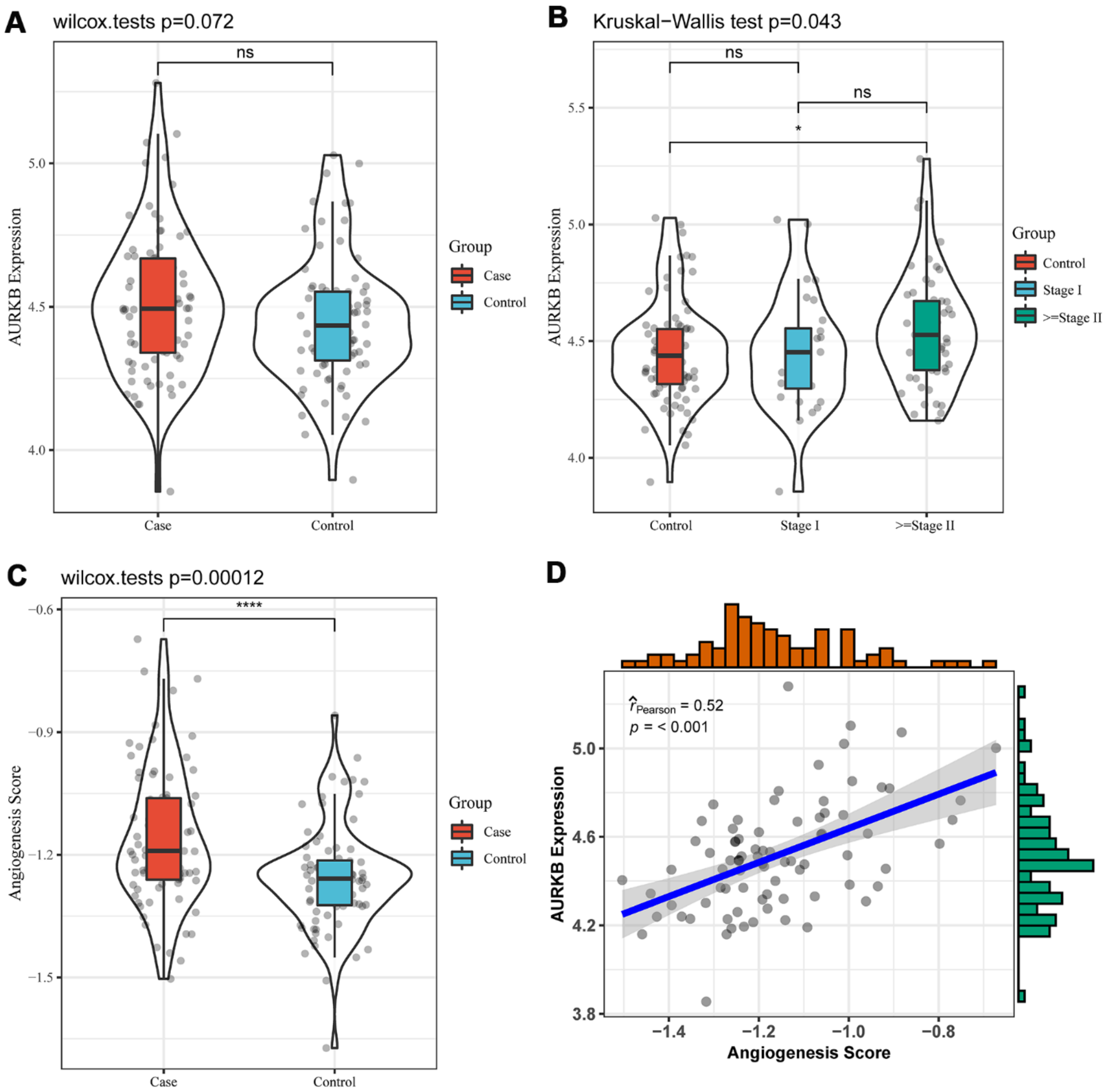

Supplementary Figure 1. The potential of AURKB as a marker in serum samples. (**A**) The expression of AURKB in tumor serum samples and normal serum samples. (**B**) The expression of AURKB in normal samples, early and advanced samples. (**C**) The angiogenesis factor scores between tumor and normal samples; (**D**) The correlation between AURKB expression and angiogenesis factor score.

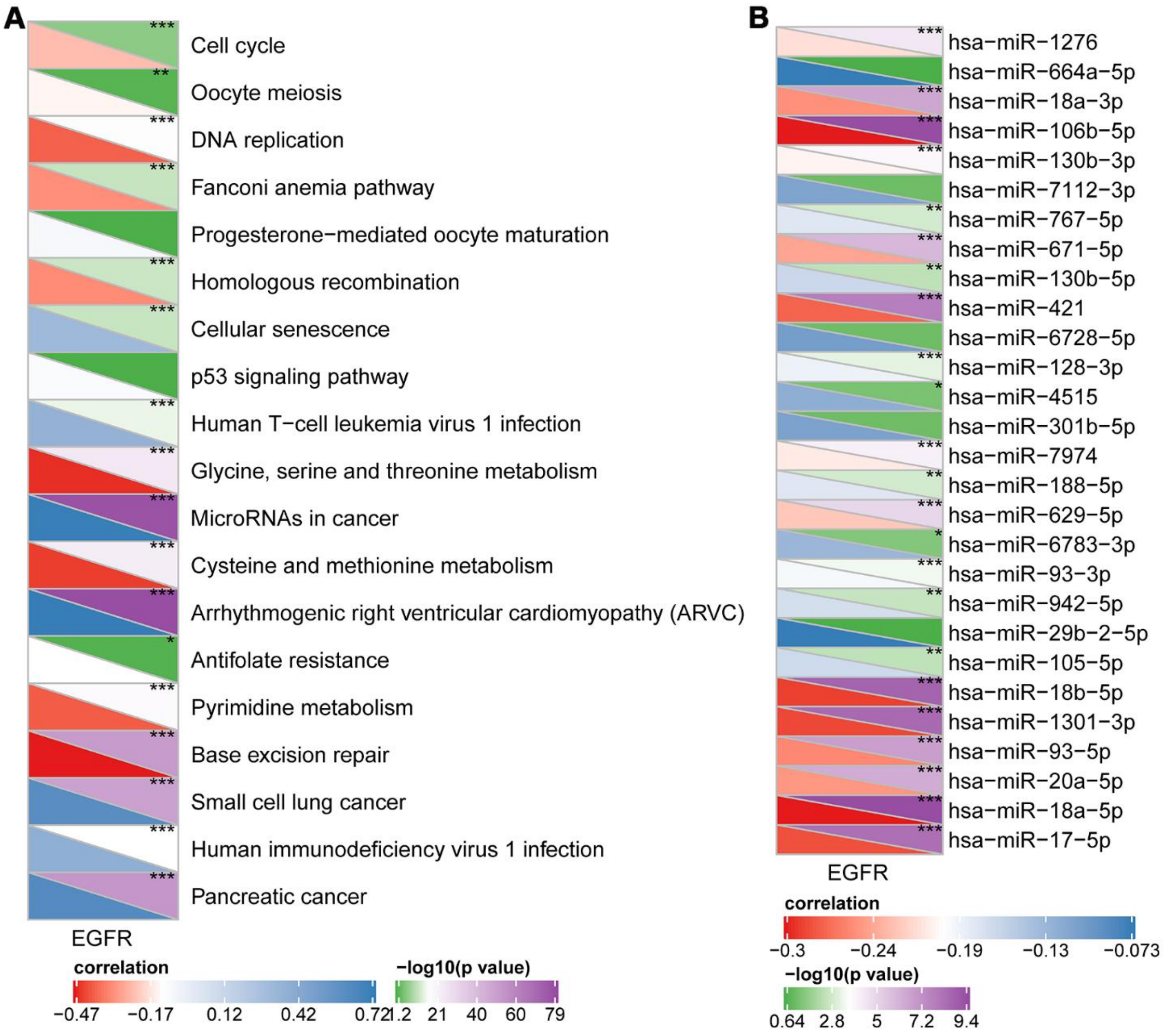

Supplementary Figure 2. The relationship between AURKB-associated miRNAs, signaling pathways and EGFR pathways. (**A**) The expression of AURKB-associated signaling pathways and the EGFR pathway. (**B**) AURKB-associated miRNAs expression is associated with EGFR pathways.

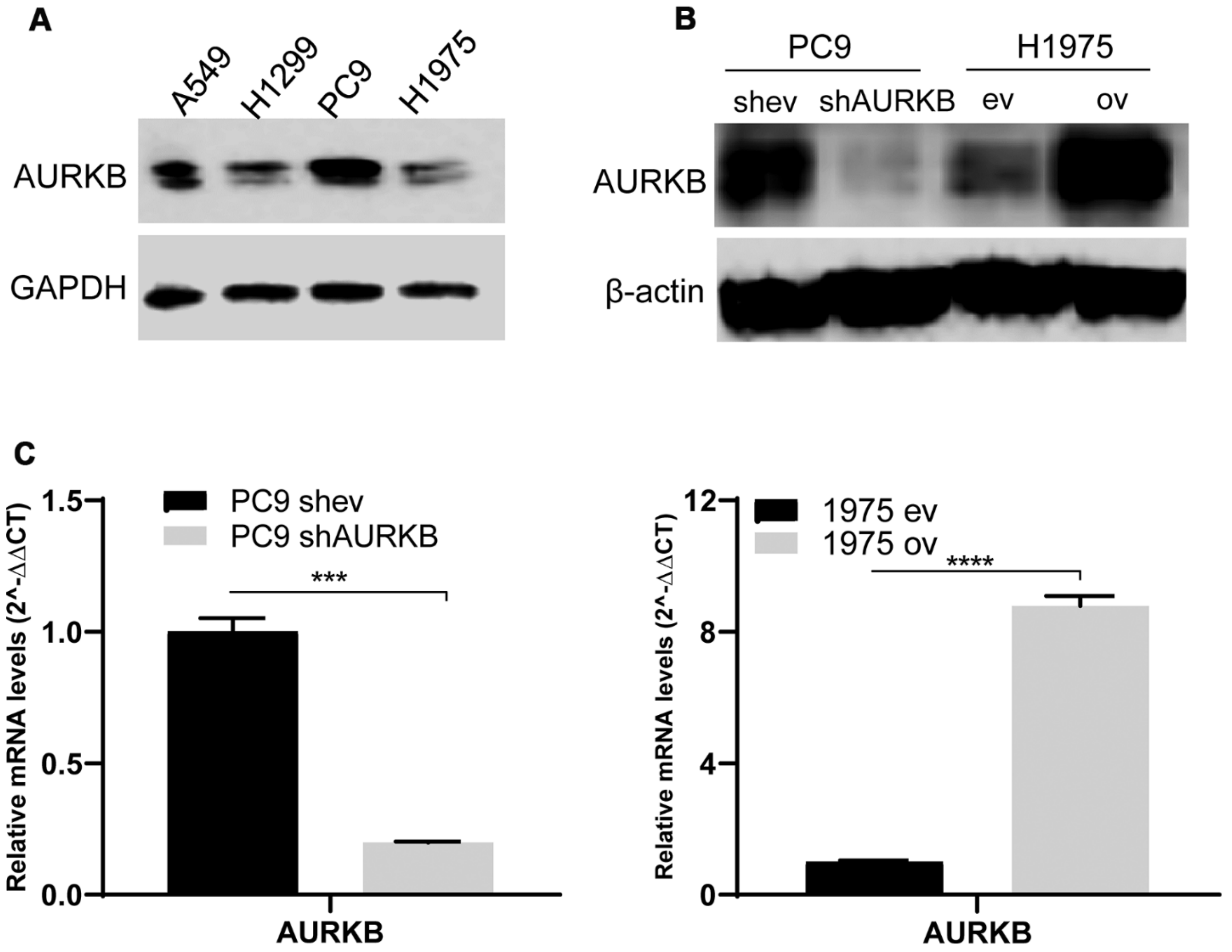

Supplementary Figure 3. Manipulation of AURKB in lung adenocarcinoma cell lines. (**A**) Western blotting of AURKB in A549, H1299, PC9, and H1975 cell lines, GAPDH as a internal control. (**B**) Western blotting of AURKB in PC9 shev, PC9 shAURKB, H1975 ev, and H1975 ov cell lines, beta-actin as a internal control. (**C**) Relative mRNA levels of AURKB in PC9 shev and PC9 shAURKB (AURKB knockdown) cells (left panel) and 1975 ev and 1975 ov (AURKB overexpression) cells (right panel) constructed using lentivirus, beta-actin served as a internal control ($2^{\wedge-\Delta\Delta CT}$ method, $p<0.001$, ***; $p<0.0001$, ****).

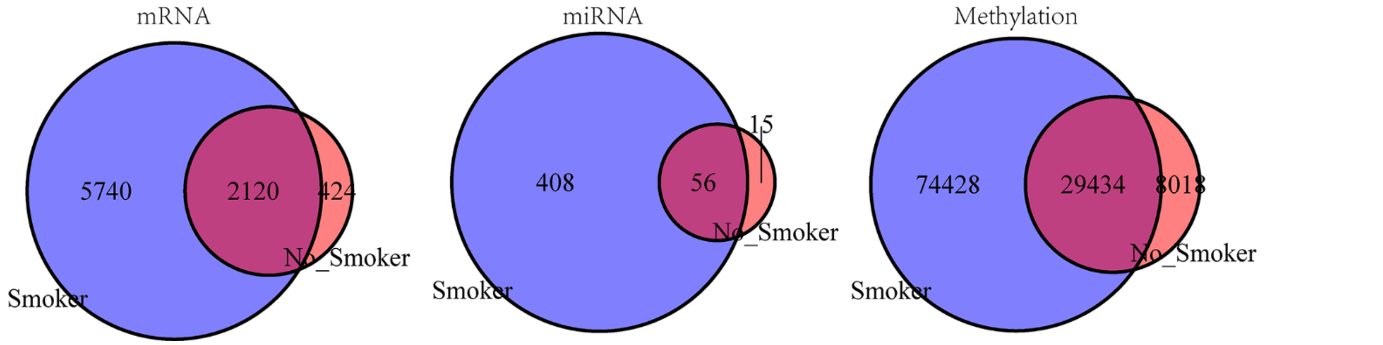

Supplementary Figure 4. Comparison of the distribution of AURKB-associated mRNAs, miRNAs, and methylation in smokers and non-smokers. The intersection of differentially expressed mRNAs (left panel), miRNAs (middle panel), and differentially methylated genes (right panel) between $AURKB^{high}$ and $AURKB^{low}$ groups in smokers and non-smokers.
